# Book Reviews

**Published:** 1810-03

**Authors:** 


### CRITICAL ANALYSIS
### OF THE
### RECENT PUBLICATIONS
### ON THE
### DIFFERENT BRANCHES OF PHYSIC, SURGERY, AND MEDICAL PHILOSOPHY.

EDINBURGH JOURNAL. No. XXI.

( Concluded from our last, pp. 151—170. )

ARTICLE 5.—*Case of a Scirrhous Ovary, in which was found an Adipose Tumour, containing Teeth and Hairs, in a Patient who died in the fifth Month of Pregnancy.* By JAMES MILLMAN COLEY, Member of the Royal College of Surgeons in London, and Surgeon in Bridgnorth.

THIS is an addition to those melancholy cases, which it is at present enough that they are faithfully recorded. In some future period, riod, an accurate comparison of the whole may lead to rational conjectures, if not to a knowledge of the nature of such præternatural appearances.

Article 6.—*On the Effects of Arsenic.* By G. N. Hill, Surgeon.

( Concluded from the last. )

In this paper, Mr. Hill gives an account of his success in lepra, syphilis, hemicrania, and tic doloreux, the phagedenic ulcer, schirrhous, and cancer. The first of these diseases is too inaccurately described to enable us to determine the success of the remedy. The second relates chiefly to those cases in which mercury has either lost its effect, or become deleterious. In many of these, a bare dereliction of that remedy proves a cure. In the phagedænic ulcer, and some of the diseases called cancerous, the remedy has been long in use, and some satisfactory cures of tic doloreux are also on record. It is no part of our wish, to undervalue Mr. Hill's labours, but there is always danger in the strong partiality to individual remedies, as we have lately had frequent occasion to remark of another mineral.

Article 7.—*Sequel to "An Apology for the Cutting-Gorget."* By W. Simmons, Surgeon at Manchester.

The only remark we shall make on this paper, is the author's proposal that the staff should be held by an assistant, whilst the beak of the gorget is introduced along the groove by the operator. The advantage proposed by this plan is, that the operator may have the use of both hands. Under such a mode of operating, we conceive the assistant should not be less ready at the business than the operator himself. In our opinion, however, most surgeons would wish to manage the staff whilst they are passing the gorget through parts so highly important.

Article 10.—*On the Connection that subsists in certain Cases between Amenorrhœa and Phthisis Pulmonalis, as Cause and Effect.* By William Shearman, M. D.

This paper contains some very useful practical inquiries, concerning a subject which very often puzzles the physician, namely whether amenorrhœa is to be considered as a primary or secondary disease. In the case here described, we confess we remain very much in suspense, but cannot help offering a hint, which we doubt not will be well received by the author. When we are introduced to a young woman about 20, delicate, but healthy, till the last five months, with a quick pulse, and many symptoms of emaciation, there is always some uncertainty whether we are made acquainted with all the probable causes of her complaint. Even if the tender passion has no share in it, we shall frequently find that some source of anxiety has gradually enfeebled all the energies of life, and excited into action certain parts, which, though previously diseased, might, under more favourable circumstances, have continued without interrupting the actions of those organs in which they are seated. These causes are carefully concealed by the patient, and sometimes sometimes unknown to, or purposely overlooked by those about her. At length the disappearance of a discharge, to which the sex are, with much propriety, very attentive, gives the first alarm; but before this, considerable mischief has taken place, and the uterus is only one of various organs whose functions are interrupted.

After this preface, we shall proceed to give the case in the author's own words.

" A young woman, aged 20 years, of a very delicate habit, but in pretty good health, had experienced about five months ago an obstruction of the menses in consequence of cold; from this time her health gradually declined, she became languid and weak, had frequent pains in her side, and occasionally a short dry cough. Her appetite was impaired, and she became rather emaciated. In the month of July I first saw her; at this time she complained of pain in the left side, occasional fits of coughing, which recurred at uncertain intervals, but generally with considerable violence night and morning. She had scarcely any expectoration, little thirst, pulse 120, appetite impaired, bowels regular, countenance and lips very pale; there had been no return of the menses since their first suppression. A blister was immediately applied to the pained part, and directed to be kept open; five grains of rubigo ferri, combined with myrrh and castor, to be given three times a-day, with infusion of chamomile flowers, and the feet to be immersed in warm water every night. The pain was removed by the blister, and it was healed up. The medicines were continued about six weeks; during this time the pain occasionally recurred, which was always soon removed by a blister. The cough was sometimes considerably better, sometimes aggravated, with trifling expectoration; her pulse was diminished to 90; her appetite improved, and her countenance looked better. I now discontinued my attendance, and have since learned, that the only remedies she afterwards used were, expectorants and blisters, occasionally, when there was cough or pain, and she *entirely omitted* all other medicines. She continued without much alteration, excepting that she gradually grew weaker, and within the last fortnight was almost confined to bed, without, however, the cough or expectoration being greater than before. On the Saturday morning previous to her death, she was seized with violent excruciating pain in the abdomen, particularly about the umbilicus, which continued, with more or less violence, for several hours; it then left her, and she seemed much in the same state as before, until the Thursday morning, October 13th, when, soon after having got out of and into bed, without assistance, she suddenly expired.

" On opening the thorax, there was found a very general adhesion of the pleura pulmonum to the pleura costalis, yet not so firmly but they might be easily separated by the fingers; there was a small quantity of fluid in the cavity, not exceeding three or four ounces; the lungs were very dense, and of a dark colour, resembling sembling the liver in appearance; portions of them, when put into water, fell to the bottom. Being cut into, some of the bronchial cells contained a purulent matter, but in small quantity, and here and there was a small excavation the size of a pea; a few small tubercles also were found, not inflamed. The external surface of all the small intestines was very red, and seemed to be studded with small red points, resembling the villous coat of the same intestines when injected. There was no appearance of gangrene, neither was there any adhesion between the different convolutions of them, nor between any of them and the peritonæum; the omentum also was extremely vascular, the mesenteric glands were enlarged and hardened in several places, the pancreas, spleen, and other viscera, were of their natural appearance.

"That the lungs were injured in a great degree, was evident from the tubercles found in them, and from the pus contained in the bronchial cells, but still the ulceration had not arisen to such a height as to be the immediate cause of death. More extensive affections than the present one are perhaps frequently recovered from, and tubercles may remain for many years with impunity, if no exciting cause of inflammation arise. Although, likewise, there were marks of inflammation upon the intestines, yet this inflammation did not immediately destroy the patient; for the pain had left her some days previous to her death, indicating a cessation of the inflammation, which, as it was not followed by gangrene, cannot be considered as the cause of death. The enlarged state of the mesenteric glands affords a presumption, that the flow of chyle into the blood-vessels might have been interrupted, but certainly there was not sufficient appearance of emaciation in the dead body, to enable us to attribute her death to this circumstance. We must look for the cause of death, not in the affection or destruction of any one particular organ, but in that state of general debility, consequent upon the interruption of a very important function in the female economy, during which state of debility, parts of the body peculiarly predisposed are very apt to take on disease. In the present patient, there appeared to exist a scrofulous diathesis, wherein the lungs and mesenteric glands are always readily susceptible of disease, from the slightest causes, and always the more readily the greater the state of weakness in the system, however induced. Every kind of weakness is accompanied with irregularity in the action of the living power; hence spasmodic contractions of various parts. This disposition to preternatural contraction is particularly observable to take place in the blood-vessels, in all cases of amenorrhœa, forming the principal symptom in what has been called chlorosis, differing from hectic fever, chiefly, in depending upon a morbid action of the vessels themselves, independent of the absorption of extraneous acrimony. This state of the vessels is perhaps at first produced by plethora, arising from a retention of a quantity of blood, which excites the larger arteries to stronger action, while yet the resistance in the extreme vessels in the uterus is not overcome, come by its increased action, long continued, exhausts the living power, and produces weakness. During this state of general weakness, and morbid action of the blood-vessels, partial inflammations are very apt to arise. No wonder, then, that the lungs, when, from constitutional predisposition, they are so susceptible of disease, should be one of the first parts to take on inflammation. If by suitable remedies the lungs are relieved, (the disposition to general inflammation still continuing from its original cause, the suppression not being removed) some other part is very readily affected; hence in the present case arose the inflamed state of the intestines, during which, all the thoracic symptoms disappeared. That this irregularity of action in the vessels, sometimes exciting symptoms of inflammation in one part, sometimes in another, entirely depends upon the suppression of the menses, is evident, from its generally ceasing upon the return of that evacuation, and from the perfect restoration of health immediately following it. No occurrence is more common than the attack of cough, pain in the side, and difficulty of breathing in females, soon *after* the obstruction of the menses, and upon their recurrence all these symptoms going off."

"Dr. Beddoes, it is urged, observes, 'In some instances, when the fox-glove has removed the hectic fever, and greatly reduced the *expectoration and cough*, the decline shall become almost imperceptible, the patient *frequently appearing chlorotic*, but being really phthisical, as the *event* most commonly, and *sometimes* dissection, has evinced.' This serves to confirm the opinion, that the pulmonary symptoms are not in these cases the original idiopathic disease. If it be objected, that this doctrine can only be applicable to incipient phthisis in *females*, I would appeal to the experience of practitioners to determine, whether, in the female sex generally, the approach of phthisis is not more insidious, and its progress more slow than in men, where we usually find the attack more marked, and immediately consequent upon some obvious occasional cause, the symptoms sooner becoming more violent, and its whole progress more rapid. That this difference in the appearance of the disease arises chiefly from its frequently being rather symptomatic than idiopathic in females, will be apparent, if we find, that when phthisis arises in them from some obvious occasional cause, and where there has *never occurred suppression of the menses*, its attack and progress are more marked and rapid, than in the male. That it is so, I think experience will justify me in asserting; and impressed with this opinion, I cannot but recommend that we should keep in view the probable dependence of the pulmonary symptoms upon the interrupted functions of the uterus, and direct our treatment accordingly, at least, until we are convinced upon trial, that the opinion itself is fallacious, and the proposed treatment inefficacious or prejudicial."

Many ingenious observations are added by Dr. Shearman, on the remedies found successful in such complaints, particularly the celebrated lebrated recipe of Dr. Griffiths, and the success with which it is sometimes attended in cases which threaten pulmonary consumption. On the whole, the paper abounds with useful practical remarks.

ARTICLE 11.—*Case of a singular Disease of the Heart.* By HENRY WISHART, Member of the Royal College of Surgeons, and one of the Surgeons of the Public Dispensary, at Edinburgh.

ANOTHER melancholy case, apparently arising from inflammation at the source of the circulation. We trust the frequent occurrence of such cases, will gradually convince every practitioner how improperly the lancet has been omitted, or how absurdly it has been dreaded in many cases of intense pain in those regions which contain viscera, the interrupted functions of which, must produce a calamitous life, if not an early death. We shall not be suspected of arraigning the practice of any individual. The commencement of the above case was much before the relator was consulted. His candour and accuracy does him equal credit. But we wish to take every opportunity of impressing on that class of our readers, who, for the most part, see diseases in their earliest stage, the importance of arresting them in the beginning, watching the progress of the patient's convalescence, and even for some years after attending carefully to every symptom, especially at periods of the year which have previously proved alarming.

---

*Surgical Observations on the constitutional Origin and Treatment of Local Diseases; and on Aneurisms.* By JOHN ABERNETHY, F. R. S. Honorary Member of the Royal Medical Society of Edinburgh, and of the Medical Societies of Paris, Philadelphia, &c. Assistant Surgeon to St. Bartholomew's Hospital, and Teacher of Anatomy and Surgery.

BY a Preface we are informed, that this work is to be considered a republication of the Author's former writings, leaving out the physiological parts. "With regard to the cases," says Mr. Abernethy, "which I consider as the valuable part of the book, I may observe, that it is not to be expected that the records of them will make so strong an impression on the minds of the readers, as the observance of them has done on that of the writer; but when the same occurrences are met with in practice, then will the impression become more vivid, and knowledge arise, as it usually does, from personal experience. If the facts contained in these volumes occurred so rarely, that others could not be expected to meet with them, their relation would be of little value. They may, however, not improperly be compared to certain species of plants, by no means uncommon, which are liable to be confounded with others by an inattentive observer; but when their discriminating characters are once pointed out, they may be so readily distinguished, collected, and examined, as to render a more minute description of them unnecessary. If diseases could, like other objects which we mean to delineate, be placed in various points of view, and illuminated at pleasure, so as to show distinctly their different parts, one accurate representation would suffice; but we see them obscurely, and as knowledge increases, it serves, like light shining from different places, to illuminate the various parts of the objects of our examination.

"For, as I have expressed it in the first edition of these writings, 'in proportion as we advance in knowledge, we are led to remark many circumstances in the progress of a disorder which had before passed without notice; but which, if known and duly attended to, would clearly point out the nature of the complaint. Hence the records of former cases are of much less value; as the symptoms about which we are now anxious to inquire, have in them been entirely overlooked.' It therefore becomes necessary, that each writer should state those circumstances to which he has been particularly attentive; nor need he further delineate the case than by a general outline, so as to render it intelligible.

"The relation of cases may be compared to the representations which an artist gives of natural objects, and which are valuable only inasmuch as they are correct or vivid delineations of reality. Such portraits, sketched by a person of dull perception, or by one whose optics are perverted by prejudice and theory, are either valueless or deceptive; and hence, perhaps, has arisen that objection to books of cases which I find to be very prevalent. In the imperfect sketches which I have laid before the public, my chief object has been to touch up, and bring into view, some parts of the subjects which have not been so clearly seen or strongly delineated by former draftsmen."

This passage shows very clearly the importance of marking with minuteness every symptom of disease, and the advantage which an experienced practitioner will derive from his previous knowledge in detecting many particulars which may be overlooked by others. But the allusion to artists very much reminds us of one failing, of which, according to their technical language, many of them are accused, viz. of being *mannerists*. Genius and correctness are, unfortunately, too seldom united. The rapidity with which the minds of some men embrace objects of the most complicated nature, is highly flattering to themselves, and not less agreeable to such learners as conceive the whole of any science may be comprized in a nut-shell. Hence the fermentation of Sydenham, the spasm of Cullen, the sthenics and asthenics of Brown have been popular, because they seemed to be comprehended without trouble, and to be, with certain modifications, applicable to most diseases. These theories are, perhaps, most dangerous to those who receive them at second hand. Sydenham described his cases with accuracy, and varied his practice according to symptoms, though he contrived some way to reconcile all this variety with his favorite theory. But his followers were so tenacious of his doctrine, that in their eagerness to conform to it, their practice for some time after their master's death was directly opposed to his.

 We

We cannot help apprehending something of the same kind in our own days. That men of experience may entertain their favourite notions, and even model those notions to the facts before them, we can easily conceive; and if their practice varies conformably, no injury will be suffered. But if doctrines, apparently simple, and applicable to all cases, are strongly impressed on young men, will there not be too much reason to fear lest the result should be an uniformity in practice, scarcely consistent with the variety of forms in which diseases present themselves in different subjects and at different seasons? We wish these suggestions to be considered in a very general view, and shall now proceed to our Remarks on the Work, which is introduced in the following manner.

"An evil, (says our Author) seems to me to have arisen from the artificial division of the healing art into the medical and surgical departments. This division has caused the attention of the physician and the surgeon to be too exclusively directed to those diseases, which custom has arbitrarily allotted to their care. The effects of local disorders upon the constitution have in consequence been too little attended to; and indeed I know of no book, to which I can refer a surgical student for a satisfactory account of those febrile and nervous affections which local disease produces, except that of Mr. Hunter. The reciprocal operation of constitutional disorders upon local diseases has obtained still less attention. To investigate more particularly some parts of these subjects, and to submit them to public notice, are the proposed objects of the present paper.

"No part of the animal body can in general be very considerably disordered, without occasioning a correspondent derangement in other parts of the system. Such disorder has been considered by Mr. Hunter as the result of universal sympathy. This consent of the whole constitution with its parts, manifests itself, in particular instances, by a greater disturbance of the functions of some organs than of those of others; and from this circumstance diseases have derived the appellations by which they are commonly distinguished. If the actions of the sanguiferous system be principally disturbed, and the temperature of the body subject to unusual variations, the disease is termed fever: if the nervous system be chiefly affected, a state of vigilance or of delirium may be produced; convulsions and tetanus take place when the functions of the muscular system are more particularly deranged. Though the disorder of particular organs thus give a character and denomination to the disease, it is sufficiently evident, in the instances adduced, that the whole constitution is disturbed; while certain parts are chiefly affected, perhaps from unknown circumstances relative to the nervous system, or from a predisposition to disorder existing in the affected parts. It seems to be ascertained, that persons of particular constitutions are predisposed to those febrile actions of the sanguiferous system, which constitute the inflammatory fever; ver; that there is a propensity to convulsions in children; and to tetanus in the inhabitants of warm climates.

"It may be a fit subject for inquiry, whether it be possible for particular organs to become affected otherwise than through the nervous system in general. Though some instances of sympathy are strange, and perhaps inexplicable, there are strong reasons for believing that the inflammatory fever, the state of vigilance and delirium, convulsions and tetanus, which arise in consequence of injuries of the limbs, are produced by irritation imparted to the brain, which, by a kind of reflected operation, occasions a greater disorder of some of the organs of the body than of others, and thus gives a character and denomination to the disease.

"That the stomach and bowels are disordered by injuries and diseases of parts of the body, has been remarked by various persons; but the subject has never been extensively surveyed, nor viewed with that accuracy of observation, which its high importance merits. It has been observed, that sprains of tendinous or ligamentous parts produce sudden sickness; and Mr. Hunter has attributed that shivering which is consequent to accidents, and attendant on some diseases, to the state of the stomach. It is known that, in some local injuries from accident or operations, the stomach has appeared to be the part principally affected. But remarks on the affections thus induced in the digestive organs have been made only in a cursory manner; and it is my intention to examine the subject more particularly. It also appears to me, that the connection of local diseases with the state of the constitution in general, is either not sufficiently understood, or not duly regarded, by the generality of practitioners; and I also mean to claim their particular attention to this subject. I shall in the first place select a case, to shew how the stomach and bowels, or, to speak yet more extensively, the digestive organs may be affected from local disorder."

It is impossible not to regret the vast scope of reflection that is forced on the reader in this short passage, without time to look about him, or any clue to direct him. Mr. Hunter is first introduced by referring the student to the largest, most profound, and in some respects, most complicated of all his works, and without the slightest direction to any passage; yet this is the only book in which will be found a "satisfactory account of those febrile and nervous affections which local disease produces." We are afterwards indeed informed, that Mr. Hunter considers such disorders as "the result of universal sympathy;" and in a Note we are further told, that it is the remote sympathy of the "patient and industrious" Mr. Hunter of which Mr. Abernethy speaks. By all this, we suppose it must be the intention of the Author to distinguish between that sympathy which shows itself by the affection of only one organ, from that which affects the whole constitution. Mr. Hunter has indeed explained himself with sufficient accuracy in this distinction. With him, the sympathy of the whole constitution tution is well enough known by the terms symptomatic and hectic fever, and the remote partial is not less understood by the affection of the head with the stomach, the stomach with the uterus, tendons, and many other parts. All this, though intelligible in Mr. Hunter, it must be confessed, is related with more brevity than its importance, as a fundamental doctrine, seems to admit. Whether it is more perspicuous in Mr. Abernethy, our Readers must determine.

A case follows, in which the operation for the omental hernia was followed by symptomatic fever of three days. In the beginning, the stomach rejected every thing that was swallowed. After copious bleeding the sickness abated; but the tongue was furred, the skin hot, and the pulse quick, with great watchfulness. The patient took small doses of Epsom salts, and other purgatives, which remained on his stomach, but produced no effect. On the fourth morning, he "felt his bowels, to use his own expression, apparently filling; and a profuse discharge ensued. A dozen copious, fetid, and black evacuations took place between five and ten o'clock, and he had several others in the course of the day; after which his appetite returned, his tongue became clean, and a sound and continued sleep succeeded."

Mr. Abernethy remarks, that on this occasion the chylopoietic organs were the parts chiefly affected. We are by no means disposed to question this, for there cannot be a doubt that in all fevers, from whatever cause, all the functions are disordered; and the great importance of those abovementioned, render them the earliest object of attention. In such a state as this, it is not to be wondered if the intestines were insensible to very powerful stimuli, nor that their sensibility returned as the system at large recovered from its symptomatic irritation.

"It is probable also, (says Mr. A.) that the restlessness and anxiety of the patient were aggravated, if not principally caused, by the state of the chylopoietic viscera; since the relief which took place in those parts on the renewal of secretions into them, certainly removed the nervous and febrile symptoms. That the discharges were the effect of secretion is proved by the absence of alimentary matter in the bowels, in consequence of the action of the purgative administered on the morning of the operation, and the abstinence both before and after that period."

For our own parts, we should be disposed to consider the disorder in the chylopoietic organs as part of that general irritation which induced disorder in every other part; and we should, for the same reason, consider the impróved condition of those organs, and their sensibility to stimuli, as the consequence of the cessation of that general irritation, that symptomatic fever, that universal sympathy, which was excited by the operation. It is scarcely less common for the brain to be affected under similar cirstances than the stomach and bowels; in short, universal sympathy is excited, and those parts suffer most which, according to the constituconstitutional peculiarity of the patient, are most easily irritated. Whilst this irritation continues, every attempt at producing the customary effects of medicine will prove abortive. If symptoms of inflammation attend, bleeding is the only remedy; after which, quiet, and the administration of such simple liquids as are found most agreeable to the patient, are all that can be attempted till the irritation subsides.

"I could relate (says Mr. Abernethy) numerous cases in support of the inferences which I have drawn from the preceding history, that local irritation acting on the nervous system may affect the digestive organs in a very serious manner, and thereby create great general disorder of the system, which is afterwards alleviated in proportion to the amendment that ensues in the state of those viscera. Such consequences of great local irritation must frequently occur to every one; it is therefore unnecessary to adduce more instances to support the opinions here delivered.

"With respect to the treatment of cases of this description it may be right to add, that the primary object should be to produce secretion from the irritable organs. In the case which has been related, and in many others recorded in this volume, the effect of secretions occurring from the disordered organs in relieving their irritable state is very manifest. In many instances opium will not prevent continual efforts to vomit, yet when by magnes. vitriolat. or purgatives administered in the form of pills and clysters, stools are procured, the vomiting ceases, the stomach retains both food and medicine, and general tranquillity of constitution is as suddenly restored."

We should rather say, that when these organs recover with the rest from the universal sympathy, their secretions will follow the customary stimuli. In the above case, Mr. A's primary object was copious bleeding, before which the stomach rejected every thing.

A Chapter, or a proposition follows, entitled, "A slighter degree of continued local irritation will produce a less violent disorder of the digestive organs." In this we meet with nothing to which we can object, excepting that some well known practical facts are delivered in a language which would lead us to suppose that the author was telling us something new. We extract the following paragraphs, to show the author's mode of describing hectic fever from large abscesses; the mode in which a common ulcer partakes of the fluctuations of health in an irritable subject; and the various sympathies attending dentition.

"If then, (says Mr. A.) vehement local irritation can produce a violent disturbance of the chylopoietic organs, it may be expected that a less degree of a similar cause will produce slighter effects of the same nature. Indeed, the foregoing case was related not merely because it seemed worthy of record by itself, but chiefly to prepare the reader for the observations which are to follow.

"This slighter degree of derangement occurs in the advanced stages of lumbar abscess, diseased joints, compound fractures, and all kinds of local disease, which impart considerable and continued irritation to the whole constitution. We also find a less important disease, as, for instance, a fretful ulcer, keep up a disorder of the system in general, and of the digestive organs in particular, which subsides as the irritable state of the ulcer diminishes. But as practitioners in general may not, perhaps, have so attentively remarked these circumstances, as to be familiarly acquainted with them, it may be useful to mention a very common occurrence, which cannot have escaped observation. I allude to the effects of the irritation of teething upon the health of children. The brain is sometimes so affected as to cause convulsions; the digestive organs are almost constantly disordered. The appetite fails; the tongue is furred; the secretions of the liver are either suspended, diminished, or vitiated. The bowels are either purged or costive, and the fæces fetid. The fæcal matter is often mixed with mucous and other secretions. There is also frequently a very troublesome cough. Such symptoms generally subside when the local irritation ceases, but sometimes the disorder of the digestive organs, thus excited, continues and disturbs the general health of the patient."

The subject of the digestive organs, of the biliary secretions, and of the alvine discharges, is continued for several pages; but whatever axioms or inferences the author may wish to draw, we confess reluctantly, that we have not been able satisfactorily to inform ourselves. There are many common remarks mixed with others which are more new; but the loose manner in which the whole is tacked together, deprives the reader of many advantages, which the experience of Mr. Abernethy must otherwise have afforded. In the midst of many conjectures on the use of the bile, we have the following passage in the form of a Note.

"In the inquiry into the probable uses of the bile, it ought to be observed, that in many persons, in whom that secretion is either for a considerable time wholly suppressed, very deficient, or much depraved, it does not appear that the nutrition of the body is defective."

In a work intended to instruct the younger practitioners, it would have been desirable, that this note should have been taken into the text and more extended. By the last limb of the sentence, is it intended we should understand, that the body being nourished as before, no inconvenience occurs to digestion from the loss of bile? or is it only meant to show, that the quantity of fat may be the same, though digestion is injured from a deficiency of bile? It is certain, that in children who live much on milk and farinacea, the stools are frequently colourless without any apparent want of health, or even of cheerfulness. On this account, we should have thought less of the remarks contained in the note, had not our author insisted, afterwards, so much on the colour of the fæces, as indicative of the state of health.

Another section devoted to this part of the subject, describes the defect of these organs as manifested by a dry tongue, furred particularly larly at the back part, pain in the epigastric region, turbid urine, &c.; in which we have still to regret the want of connection or arrangement, whilst we cannot but be pleased with many of the remarks.

We are next presented with some paragraphs, superscribed, "Occasional effects of disorder of the digestive organs."

This relates principally to the treatment; the plan of which may be easily conceived, as we have hitherto heard of little but diseases of the digestive organs. Mild aperients with stomachic bitters, and occasionally mercury in small doses.

"When the state of the health required it, says Mr. A. or the disease did not yield to the treatment which I have described, I have referred the case to the physician; under whose direction benefit has been obtained by medicines of more activity than those which I had ventured to recommend, conjoined with tonics, and those medicines which are usually termed nervous.

"In investigating the treatment of these disorders, it is necessary to ascertain, not only what medicine is beneficial, but also what change it produces in the circumstances of the disorder. The administration of a medicine may in one case be succeeded by a discharge of bile, and a striking relief from long continued and distressful feelings: yet the same medicine may be given in many other instances without the same consequence. Was the change, then, in this instance, accidental? Or must it be attributed to some unnoticed peculiarity in the disease or constitution?

"I have generally explained to the patients, the objects which I had in view, in correcting disorders of the digestive organs, by saying that there are three things which I consider as right and necessary to the cure of disorder. First, that the stomach should thoroughly digest all the food that is put into it. The patient perceiving the necessity of obtaining this end, becomes attentive to his diet, and observes the effect which the quantity and quality of his food and medicines have upon his feelings, and the apparent powers of his stomach. Secondly, that the residue of the food should be daily discharged from the bowels: here too, the patient apprized of the design, notes what kind and dose of purgative medicine best effect the intention; and whether it answers better if taken at once, or at intervals. Thirdly, that the secretion of bile should be right, both with respect to quantity and quality. In cases wherein the secretion of bile has been for a long time deficient or faulty, I recommend, as I have said, unirritating and undebilitating doses of mercury to be taken every second or third night, till the stools become of a rhubarb colour. This mode of exhibiting the medicine has at least the advantage of being innocent, and if months elapse before the object is accomplished, we cannot wonder at the tardiness of the cure, when we consider the probable duration of the disorder, prior to our attempts to correct it. The patient is relieved in proportion as the end is accomplished, which feelingly induces him to persevere in such innocent measures. By thus engaging the co-operation of the patient, the practitioner titioner will, in my opinion, derive considerable advantage in the treatment of the case."

By this passage, we learn that Mr. Abernethy consigns his more stubborn cases to the physician. Some lighter cases it appears are relieved almost instantly; on these he expresses a doubt, whether he is to impute the change to "accident, or some unnoticed peculiarity in the disease or constitution." We are concerned to see such a waste of language under all the formality of observation! What is accident, we would ask? — Does true philosophy admit any such thing as accident? And if pathology indulges herself in such an expression, does she mean any thing but "some unnoticed peculiarity in the disease or constitution." Why then this distinction without a difference: And if, as we are told on other occasions, months elapse before the object is accomplished under this innocent mode of treatment; do we not know how generally such ailments relieve themselves, and how ready that class of patients, who love to "co-operate" with their medical adviser, are to fancy themselves better, under a plan which they suggest or approve.

The work now takes the form of Sections, containing "cases illustrative of the author's doctrine. The first is on nervous and muscular disorders." These are chiefly paralytic diseases, resembling such as arise from a distorted spine or carious spinal processes. In some of these the author found great advantage from such remedies as restored the digestive organs, or such a plan of life as proved the means of generally invigorating the patient. These Cases may be read with great advantage by the younger practitioner. The habit of applying issues or blisters indiscriminately in all such cases, we have often seen reason to regret, and excepting where the disease seems connected with chronic inflammation, we never saw much benefit derived from them. We would however, remark, that as Mr. Abernethy finds his plan succeed sometimes suddenly, sometimes after months, so Mr. Pott found his caustics always succeed, sometimes early, sometimes after more than a twelvemonth.—Let these foibles, in distinguished characters, teach us all to be suspicious of the suggestions of our own fancy!—There is a short note which we cannot omit.

"I have seen several cases which induce me to believe that the weakness of the sphincter vesicæ, which occasions young persons to void their urine during sleep, very frequently arises from the same cause."

Thus a process which takes place only during sleep, arises from a weakness in the sphincter vesicæ in subjects who, whilst awake, can retain their urine without difficulty. We shall only hint at another very eminent surgeon, who seems rather disposed to cure all diseases, slow fevers among the rest, by setting the urinary organs to rights, just as Mr. Abernethy would cure a defect in the urinary organs by improving the digestion, and thus relieving his patient of a low fever.

"I think, says Mr. Abernethy, that local irritation may disorder order the digestive organs; which disorder continuing, and aggravating the affection of the sensorium, may possibly lead to the production of tetanus, at a time when the wound is no longer irritable. In four cases of tetanus, in which I had an opportunity of inquiring into the state of the bowels, the evacuations from them were not like fæces. I wish to propose, in investigating the cause of tetanus, as a question, What is the state of the bowels between the infliction of the injury and the occurrence of that dreadful malady?*

On this passage we shall only remark, that the reader cannot be directed wrong in his practice. The *local* disease he will of course attend to, nor do we suppose he will be inattentive to any error in the digestive organs. The question proposed at the close of the paragraph is we apprehend intended for other climates, where lock-jaw is more common. In this country it is so little expected, and when it occurs, is so generally after the appearance of a wound is mending, that we shall scarcely persuade every patient to watch the colour of his fæces during the whole period. In warmer climates the event is more common and more rapid. If in these an alteration should be found in the appearance of the fæces, will it not be rather suspected, that the digestive functions have suffered in common with the other parts of the system, than that they have been the immediate cause of lock-jaw?

In the next Section, the same inquiries and suggestions are continued "on the effect of disorders of digestive organs attending injuries of the head." The 3d section is on "undefined and undenominated diseases arising from disorder of the constitution;" the 4th, "on more defined diseases, as carbuncle and scrofula, arising from disorders of the constitution;" the 5th, "on diseases of various glands, arising from disorders of the constitution;" the 6th, "on disorders of parts which have a continuity of surface with the alimentary canal." As all these are directed to the same object, our readers will not expect us to be very particular in detailing the various cases by which they are illustrated. It is enough to say, that

* "Such cases as I have related, with others that it would be foreign to my present purpose to mention, have impressed the opinion on my mind, that disorders of the digestive organs may originally cause, or may secondarily aggravate a nervous disorder; and produce, as has been 'mentioned, in the nervous system, a diminution of the functions of the brain, or a state of excitation causing delirium, partial nervous inactivity, and insensibility; or the opposite state of irritation and pain; in the muscular system, weakness, tremors, and palsy; or the contrary affections of spasms and convulsions.' Could these circumstances be proved, it would be scarcely necessary to add, that those painful affections of parts, to which perhaps some predisposition exists, may be excited in a similar manner; such as gout and rheumatism. Indeed, rheumatic pains are very usually concomitant upon that state of constitution, which existed in the patients whose cases I am relating."

there there are few disorders, or few parts disordered, that Mr. A. has not relieved by small doses of mercury, stomachic remedies, and gentle purgatives. Nor do we see any necessity for this division of the *seat* of a disease any more than of the disease itself.

The last mentioned Section, on disorders of parts which have a continuity of surface with the alimentary canal, begins with diseases of the œsophagus, the Eustachian tube, or trumpet, as it is here called, and the nose; after which we have accounts of diseases in the eye, and on the common skin. It may be urged, that as the skin covers all these parts, the continuity of surface is known to exist. But is not such equally the case with every other part of the body? Why therefore any distinction on the score of continuity? In this respect we are ready to make large allowances for Mr. Abernethy's various engagements, and for that inattention to trifles which is sometimes thought characteristic of genius. But we cannot so easily excuse his printer. The following is the order of the Sections as they stand in the book. S. I. II. III. V. IV. V. V. This has sometimes increased the difficulty of making a clear analysis of the work, and will, we trust, be admitted among our excuses.

The last Section is the most interesting, as it contains what information the author has "obtained by dissection, relative to the *causation* of other diseases by those of the digestive organs." This abounds with many useful passages, some of which we would willingly extract, had we not already exceeded our limit, and did not a new subject still remain for our notice.

This division of the work begins like the former, with honourable mention of Mr. Hunter. It is however shown, that the great improvement made by that gentleman in the operation for the aneurism, appears, if not imperfect, at least to require the management of an artist equal to the inventor. The recurrence of hæmorrhage after the single ligature of the artery, is accounted for by Mr. Abernethy, in a satisfactory manner, as far as his mode of conducting the operation extends. His remarks on the plans proposed and adopted for improving the operation are still more satisfactory, and we are ready to admit the propriety of dividing the artery between the two proposed ligatures. But there is another name introduced in a Note, to which we conceive too little attention is paid. To explain ourselves the better, we shall extract the note and the passage connected with it.

"As large arteries, says Mr. Abernethy, do not ulcerate when they are tied upon the surface of a stump after amputation, it occurred to me that it would be right to tie them, in case of aneurism, as nearly as possible in the same manner and under the same circumstances. The large vessels on the surface of the stump continue to possess all their natural surrounding connexions, whilst they are left in a lax state, in consequence of their division.

"To accomplish this object in cases of aneurism, I propose that the operation should be performed in the following manner:—

The

The operator should divide the immediate coverings of the artery, till he has fairly exposed its surface. When he can touch the bare vessel, he will not, I believe, find any difficulty in separating from it, by means of his finger and thumb, or the blunt edge of an aneurismal needle, the cellular substance that connects it to the contiguous parts. This part of the operation is not painful, and should be performed slowly. The firm sides of the vessel enables the surgeon clearly to distinguish its surface, and by keeping the finger in exact contact with it, a passage may be made completely round the artery. Care should be taken not to elevate the artery more than can be possibly avoided, because the artery would be stretched in its longitudinal direction by so doing; and care should also be taken not to injure the contiguous veins or nerves. When the operator has thus gently insinuated his finger between the vessel and its surrounding connexions, so that an inch of its surface is every where exposed, two ligatures may be put under it one of which is to be carried upwards, and the other downwards, as far as the artery is detached, and then tied as firmly as possible. The artery should then be divided by a probe-jointed bistoury in the interspace between the two ligatures, but nearer to the lower ligature than to the upper one.

"In my opinion, large arteries should always be tied with moderately thick ligatures, because we may then draw the noose as tightly as possible, without apprehension of cutting or tearing the coats of the vessel, or of breaking the ligature. The latter occurrence would in many cases prove a very embarrassing circumstance, and it might be very injurious on account of the jerk communicated to the artery to a considerable distance. Also, when an artery is tied with a thick ligature, the compression made by it is not so great as to produce a speedy mortification and separation of the end of the vessel, so that the ligature remains, in general, a fortnight before it is detached, and therefore, time is allowed for the consolidation of the sides of the vessel prior to its separation.

"Doctor Jones, whose numerous and accurate experiments have thrown much light upon the natural means by which hæmorrhages are suppressed, thinks that the ligatures should be round and firm; because such cords are most likely to cut the internal coats of the artery. I am solicitous that they should be strong and moderately large; because, as far as I have remarked, large ligatures remain longest on the arteries before they are detached; and in examining the stumps of patients who have died after amputation, I have frequently seen the sides of the artery unclosed, even though the ligatures have fallen off from them."

In this extract, we find Mr. Abernethy expressing a wish, that the arteries should preserve all their natural surrounding connections. The advantage of this was long ago suggested in our Journal. Dr. Jones's experiments have since shown, that the preservation of this surrounding connection is of less importance, than the figure and mode of applying the ligatures. Mr. Abernethey indeed deed says, that the ligatures should be moderately thick that we may have no apprehension among other events of "tearing the coats of the vessel." Yet "Dr. Jones, whose numerous and accurate experiments have thrown so much light on the subject, thinks they should be round and firm, because such are most likely to cut the internal coats of the artery." Now, we very much wish Dr. Jones had been more attended to in this disquisition. That equally indefatigable and ingenious gentleman found, that if the internal coats of the artery are divided, the consequence is a process of nature for the obliteration of the vessel, though it should remain undivided, and even retain its permeability. This most important discovery of Dr. Jones, we cannot but regret to see entirely overlooked. It is further to be remarked, that the same gentleman proved experimentally, that if an artery is divided obliquely and transversely less than one fourth of its diameter, then the injury may be restored; but if more than one fourth of the diameter was divided, it was found that ulceration took place, in order to make a complete division, the object of which appears to be, that the artery may have the advantage of retracting, and also that the process may be set up which is found to follow a complete division of the internal coats, namely, the adhesive inflammation, by which the cavity is obliterated. All these considerations from an author such as Dr. Jones is here described, are, we think, worthy of much more attention than is paid them. Is it not possible that the very mode of tying the arteries, by making an imperfect division of their internal coats, may have produced ulceration, when a complete division might have induced adhesion and obliteration of their cavities? In the only successful case, we are informed, that Mr. Abernethy passed two "*moderately thick* ligatures, tying them as *firmly* as he could." It is much to be wished, that mention had been made, whether the ligatures were round or flat.

In all other repects we are pleased with Mr. Abernethy's courage in undertaking, and judgment in conducting such important operations; but in future descriptions of dissections, after such events, we hope more attention will be paid to the result of those invaluabie experiments, for which we are so much indebted to Dr. Jones.*

---

* For an account of Dr. Jones's labours, and our remarks on them, see our Journal, vols. xv. and xvi. And for the advantage of preserving the surrounding vessels by which an artery is supported, see our Journal, vol. vi, p. 546, note, and the passage in the text to which it refers.

Udiometrical,

